# Subcutaneous Injections of Ergotine in Uterine Fibroids

**Published:** 1873-03

**Authors:** 


Subcutaneous Injections of Ergotine in Uterine Fibroids. Dr. Hildebrandt, in the *Gazette Hebdomadaire and Berliner Klinische Wochenschrift,* 1872, gives a number of cases of fibrous tumors of the uterus successfully treated by the subcutaneous injection of ergotine. The remedy was used in the first instance for the purpose of arresting the frequent and abundant hemorrhages consequent upon the presence of the tumor, and for the purpose of compressing, by the uterine contractions, the fibroid through the cervix, and thus facilitating surgical interference. The injections were made under the integument of the abdomen immediately over the tumor. The solution employed consisted of 3 parts of ergotine, 7.5 parts of glycerine, and 7.5 parts of water. The injections were used daily for two weeks, and were discontinued upon the appearance of menstruation, and again renewed upon its cessation. The tumor gradually subsided, week by week, and finally disappeared. Fifteen weeks were occupied in the entire treatment, and the results were so satisfactory and unexpected that the treatment was repeated in a number of similar cases, and generally with the happiest results.

**Do not forget to address "Herald Publishing Company, Proprietors of the Atlanta Medical and Surgical Journal."**

